# Description of *Pella tianmuensis* sp. n. from eastern China (Coleoptera, Staphylinidae, Aleocharinae)

**DOI:** 10.3897/zookeys.539.6256

**Published:** 2015-11-23

**Authors:** Zhu-Qi Yan, Li-Zhen Li

**Affiliations:** 1 Department of Biology, College of Life and Environmental Sciences, Shanghai Normal University, 100 Guilin Road, Xuhui District, Shanghai 200234, P. R. China

**Keywords:** Coleoptera, Staphylinidae, Aleocharinae, *Pella*, China

## Abstract

*Pella tianmuensis* **sp. n.**, a myrmecophile associated with Lasius (Dendrolasius) spathepus Wheeler, 1910 in West Tianmushan Natural Reserve, Zhejiang, is described, illustrated and distinguished from its congeners.

## Introduction

The genus *Pella* Stephens, 1833 was previously represented by 63 species (Hlaváč et al. 2011, Song and Li 2013, Zheng and Zhao 2014), nine of which have been reported from China. In 2014, our team surveyed the staphylinid fauna of the West Tianmushan (Zhejiang, East China), and collected a series of an unidentified *Pella* species from a colony of the species of Lasius (Dendrolasius) spathepus Wheeler, 1910. An examination of this material revealed that the *Pella* species was undescribed.

## Material and methods

Specimens were killed with ethyl acetate and preserved in 75% ethanol before dissection. Photos of the *habitus* were taken with a Canon EOS 70D with an MP-E 65 mm macro photo lens. Head length was measured from the clypeal anterior marginto the occipital constriction; elytral length at the suture from the apex of the scutellum to the elytral posterior margin.

The following abbreviations are used in the text: BL—body length, from the anterior margin of the labrum to the abdominal apex of tergite VIII; FBL—forebody length, from the clypeal anterior margin to the posterior margin of elytra; PL—length of the pronotum along midline; HW—width of the head across the eyes; PW—maximum width of the pronotum; EL—length of elytra from the apex of the scutellum to the posterior margin of the elytra; EW—maximum width of the elytra; SL—length of elytral suture.

All the types are deposited in the Insect Collection of Shanghai Normal University, Shanghai, China (SNUC).

## Taxonomy

### Pella tianmuensis sp. n.

Taxon classificationAnimaliaColeopteraStaphylinidae

http://zoobank.org/DFC82D87-3AEF-47FB-9B4A-2EEAA6CD12E6

Fig. 1

#### Diagnosis.

The new species is characterized by dark coloration of body, bicoloured elytra (yellowish maculation extending from humeral angles to mesal area), absence of a sexual dimorphism of the head, a basally curved and apically obtuse (lateral view) ventral process of the aedeagus, and a pronounced and long crista apicalis of the aedeagus.

#### Type material

(17 ♂♂, 27 ♀♀). Holotype: 1 ♂, labelled ‘China: Zhejiang Prov., Lin’an City, W. Tianmushan (西天目山), nr. Kaishanlaodian (开山老殿), 30°20'45"N; 119°25'34"W, alt. 1200 m, 30.v.2014, Xiao-Bin Song & Liang Tang leg. // HOLOTYPE [red], *Pella tianmuensis* sp. n., Yan & Li det. 2015, SNUC’. Paratypes: 16 ♂♂, 27 ♀♀, same label data as holotype, all bearing the following label: ‘PARATYPE [yellow], *Pella tianmuensis* sp. n., Yan & Li det. 2015, SNUC’.

#### Description.

**Figure 1.**
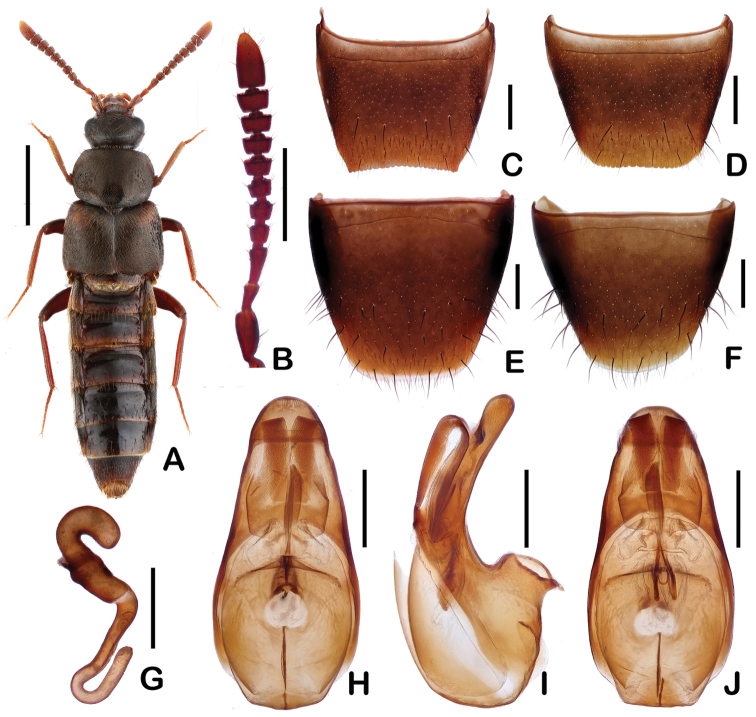
*Pella tianmuensis* sp. n. **A** Dorsal *habitus* **B** Antenna **C** Male tergite VIII **D** Female tergite VIII **E** Male sternite VIII **F** Female sternite VIII **G** Spermatheca **H** Aedeagus, in ventral view **I** Aedeagus, in lateral view **J** Aedeagus, in dorsal view. Scale bars: 2.0 mm (**A**); 0.5 mm (**B**); 0.2 mm (**C–J**).

Body (Fig. 1A) length: 4.56–6.60 mm. Coloration: fore body black; elytra bicoloured, with yellowish maculation extending from humeral angles to mesal area; abdomen brownish-black, with posterior margins of segments yellowish-brown; legs and antennae dull-red.

Head (Fig. 1A) widest anteriorly; surface finely reticulate, covered with short golden setae; antennomeres VI–X distinctly transverse (Fig. 1B). Pronotum (Fig. 1A) 1.28 times as wide as long and 1.40 times as wide as head; widest approximately in anterior third, narrowed posteriorly; surface covered with short golden setae; hypomera fully visible in lateral view. Elytra (Fig. 1A) approximately 1.08 times as long as pronotum; covered with short golden setae; humeral angle with one macroseta. Hind wings fully developed. Abdomen (Fig. 1A) widest at segments III–IV; surface with transverse microsculpture.

Male. Tergite VIII (Fig. 1C) with posterior margin slightly emarginate, its emarginated apex weakly serrate; sternite VIII (Fig. 1E) with posterior margin rounded; median lobe of the aedeagus (Figs 1H–J) cone-shaped in ventral view; ventral process of aedeagus curved at base, obtuse at apex in lateral view; copulatory piece as in Fig. 1J.

Female: Tergite VIII (Fig. 1D) with posterior margin truncate and weakly crenate; sternite VIII (Fig. 1F) with 12 or 13 pairs of macrosetae. Spermatheca (Fig. 1G) coiled three times.

#### Measurements.

BL: 4.56–6.56; FBL: 2.19–2.67; HW: 0.87–0.93; PL: 0.92–1.01; PW: 1.22–1.32; EL: 1.02–1.18; EW: 1.45–1.54; SL: 0.79–0.82.

#### Biological notes.

Most material of the new species was taken by sifting mixed leaf litter around the nest of Lasius (Dendrolasius) spathepus, together with three species of *Homoeusa* Kraatz, 1858 and with *Dendrolasiophilus monstrotibialis* (Hlaváč, Sugaya & Zhou, 2002). At least three *Pella* and some *Homoeusa* beetles were observed walking along the ant trails. Approximately five *Pella* individuals were observed eating dead caterpillars together with *Lasius* workers.

#### Remarks.

Based on the size of eyes, the shapes of the pronotum, the bicolored elytra, and the morphology of the aedeagal median lobe, *Pella tianmuensis* belongs to the *Pella cognata* group, of which four species are known from China: *Pella kishimotoi* Maruyama, 2006, *Pella sichuanensis* Zheng & Zhao, 2014, *Pella puetzi* Assing, 2009, and *Pella maoershanensis* Song & Li, 2013. The new species is distinguished from *Pella kishimotoi* by the broader and shorter ventral process of the aedeagus in ventral view; from *Pella sichuanensis* by the darker color of the body, and by the length of the elytra slightly exceeding that of the pronotum (*Pella sichuanensis*: EL/PL= 0.86); from *Pella puetzi* and *Pella maoershanensis* by the absence of a sexual dimorphism of the head and the different shape of the ventral process of the aedeagus, especially in lateral view.

#### Etymology.

The specific epithet is derived from the type locality.

## Supplementary Material

XML Treatment for Pella tianmuensis
